# Factors associated with exclusive breastfeeding practices among mothers in Mauritania: further analysis from 2019-2021 Mauritania demographic and health survey

**DOI:** 10.1186/s12889-025-22992-x

**Published:** 2026-01-22

**Authors:** Albert Apotele Nyaaba, Louis Kobina Dadzie, Jennifer Ayton, Emily Hansen, Augustina Dechegme Achigibah

**Affiliations:** 1 School of Social Sciences, University of Tasmania, Tasmania, Private Bag 22, Hobart, TAS 7001 Australia; 2 Centre for Justice, Queensland University of Technology, Queensland, Australia; 3 Department of Population and Health, University of Cape Coast, Cape Coast, Ghana; 4 Cape Coast Teaching Hospital, Cape Coast, Ghana; 5 Tasmanian School of Medicine, College of Health and Medicine, University of Tasmania, Private Bag 135, Hobart, TAS, Hobart, 700 Australia; 6 Department of Work and Social Psychology, Faculty of Psychology and Neuroscience, Maastricht University, Maastricht, The Netherlands

**Keywords:** Breastfeeding, Exclusive breastfeeding, Infant, Mothers, Factors, DHS, Mauritania

## Abstract

**Introduction:**

Exclusive Breastfeeding (EBF) confers health benefits for infants, mothers, and society. Despite this, EBF rates are low in Mauritania, and studies on factors influencing EBF are scarce. This study aims to identify factors associated with EBF practices among mothers.

**Method:**

The study applied secondary analysis on a nationally representative sample of 1,090 mother and child pairs from the 2019–2021 Islamic Republic of Mauritania Demographic and Health Cross-sectional Survey. The factors of EBF practices among mothers were identified using a bivariate and binary logistic regression. The result is presented with a 95% confidence interval using odds ratio (OR).

**Results:**

The prevalence of EBF among infants under 6 months was 43.3% [0.41, 0.47]. Children aged 2 months were less likely (OR = 0.41; CI [0.20, 0.82]) to be exclusively breastfed compared to those of 0 months. Working mothers were less likely (OR = 0.63; CI [0.41, 0.97]) to exclusively breastfeed their infants compared to mothers who were not working. Mothers residing in rural areas have an increased likelihood (OR = 2.21; CI [1.46, 3.34]) of EBF compared with urban areas.

**Conclusion:**

The proportion of infants who are exclusively breastfed remains low in Mauritania. The child’s age, wealth status, maternal occupation, region, and place of residence were associated with EBF practices. To improve EBF, creating breastfeeding-friendly workplaces, extending maternity leave, and enhancing maternal health clinics and health extension programmes in various regions are suggested.

## Introduction

The Global Nutrition Report 2022 describes the Islamic Republic of Mauritania as a country on track to meet the exclusive breastfeeding (EBF) target, with 40.3% of infants aged 0 to 5 months exclusively breastfed [1]. However, according to the most recent Demographic and Health Survey (DHS) report, only 41% of children were exclusively breastfed up to the first 6 months of life (only received breastmilk) (up from the previously reported 40% in 2016/17). Some 18% take water in addition to breast milk, while 13% consume complementary foods [2]. This suggests that the rate has essentially not changed over the past few years as such, the country may not be on track as described, considering that it is still far from both national (75% by 2025) and global targets of 70% by 2030 [3]. Despite the well-documented short- and long-term health, psychological and economic benefits of EBF, challenges still remain [4, 5]. The WHO/UNICEF describes adequate infant feeding as including the practice of EBF. WHO/UNICEF guidelines recommend early initiation (within 24 hours of birth) and EBF (only breast milk for the first 6 months) [6].

Mauritania has one of the highest infant mortality rates in Africa, with 49.95 per 1,000 live births, above the global rate of 28.9 in 2018, as well as the rates of 30 in Rwanda and 29 in neighbouring Senegal [7]. The Sustainable Development Goal (SDG) 3 target 3.2 requires reducing under-five mortality (death of a child before age 5) to 25 deaths per 1000 live births by 2030 [8]. It is worth noting that Infant mortality accounts for 75% of under-five deaths globally [9]. In low-income countries such as Bangladesh and Pakistan, EBF practices have been found to reduce infant mortality rates by 13% [10, 11], and 10% globally [12] and Mauritania may not be an exception. The quest to reduce infant and under-five mortality, with a focus on infant feeding, begins with promoting, protecting and supporting breastfeeding. This includes EBF for the first 6 months [13]. Programs such as the National Nutrition Development Plan (PNDN) (2005) and the National Nutrition Technical Committee (2008) have been implemented in Mauritania [14, 15] to improve infant and young child nutrition including EBF. Infant and Young Child Nutrition (IYCN)-related activities are carried out within six divisions or sections within the Ministry of Health, the Ministry of Agriculture, and the Ministry for the Protection of Women, Children, and Families. In addition to the governmental and international organizations working on IYCN activities in Mauritania, more than 50 local Non-governmental Organizations (NGOs) collaborated on a large-scale nutrition project [16], demonstrating strong local support for these nutrition activities [14].

Despite this, EBF rates are low in Mauritania. Evidence suggests that children who are not exclusively breastfed for the first six months are at greater risk of diarrhoea and pneumonia, the two leading causes of childhood death globally [17, 18]. A review of the literature shows that breastfeeding is socially patterned [19] and the decisions and behaviours related to breastfeeding are influenced over time by various historical, economic, cultural, and maternal and infant factors that work at different levels [20]. A significant body of literature has identified various factors that influence the pattern of EBF practices categorised into infant, maternal, household, and health service-related characteristics [11, 21, 22]. A prior study identified factors linked to the early initiation of breastfeeding in Mauritania, including birth order, place of residence, place of delivery, antenatal visits, work status, and wealth index [23]. Other research examining factors affecting infant feeding in North Africa, including Mauritania, highlighted maternal education and the child’s age [24, 25]. However, scarce literature exists on factors influencing EBF in Mauritania, and geography and context are critical to health disparities. Population-level research on the factors influencing EBF in Mauritania will guide strategies, policies and programmes designed to achieve the national and global targets for EBF. It is against this backdrop that this study seeks to investigate the prevalence of and factors associated with EBF in Mauritania.

## Methodology

### Data source and design

We conducted a secondary analysis of the latest Islamic Republic of Mauritania Demographic and Health Survey (MDHS) 2019–2021 with a sample size of *N* = 1,090. The MDHS is part of the Demographic and Health Survey (DHS) program. The DHS is a representative population database that houses over 400 surveys. It is conducted once every five years to collect high-quality data on various indicators such as maternal and child health, reproductive health and nutritional status, fertility levels, and mortality. The MDHS data was collected in two phases due to the COVID-19 pandemic. Phase one started on the 27th of November 2019 but was stopped in mid-March 2020. The resumption began in March 2021. The survey was cross-sectional and included 11,658 households. Further details of the survey and selection of participants can be found elsewhere [25, 26]. Data is freely accessible and can be downloaded after registration on the website.

### Setting

The Islamic Republic of Mauritania is in Western Africa, bordering the North Atlantic Ocean between Senegal and Western Sahara. It comprises of 15 administrative divisions (regions) with an estimated 4.2 million residents, 36% of whom are under age 15, a literacy rate of 50%, and a population density of roughly 3/km2. More than half of the population (57.7%) lives in urban regions surrounding the coastal capital Nouakchott, with tiny clusters along the southern borders of Mali and Senegal. In 2018, the per capita GDP was predicted to be $US 706 million [27]. Through the strategic framework, 2001–2015 poverty levels were reduced to 31% of the people living below the poverty line in 2014 [28].

### Sampling

The survey was intended to sample 12,120 households across the country (5,670 households in 189 primary units in urban regions and 6,450 households in 215 primary units in rural areas). According to the regions of the country, the sample is divided among the following 14 study areas: Hodh Charghy, Hodh Gharby, Assaba, Gorgol, Brakna, Trarza, Adrar, Dakhlet Nouadhibou, Tagant, Guidimagha, Tiris Zemour and Inchiri, Nouakchott North, Nouakchott West, and Nouakchott South. The two regions of Tiris Zemour and Inchiri were merged to form a dominion because of how few people lived there. The region of Nouakchott was split into three sections based on the regions of the capital because of its vast population: Nouakchott North, Nouakchott West, and Nouakchott South. Two strata—the urban area stratum and the rural area stratum—were established in each research region (except Nouakchott, which is thought to have no rural portion).

A three-stage, stratified area survey was used. In the first stage, census districts (DR) or primary units (PU) were systematically selected from a list of DRs from the Master Sample (EM) kept by the Office National de la Statistique/National Office of Statistics (ONS) and based on the General Population and Housing Census (RGPH) in 2013 with a probability proportional to their household size. It was required to draw extra PUs from the original base of the RGPH 2013 for the areas of Adrar, Tagant, Guidimagha, and Inchiri because the EM lacked sufficient PUs in all of the regions to meet the needs of the EDSM. 404 PUs were drawn in total. A thorough enumeration and segmentation were performed on the EM in 2014 to produce secondary units (SU) within each PU/DR. In both rural and urban settings, a SU is commonly equivalent to a locality or portion of a locality, with an average of 10 to 15 families per SU. In the second stage, 3 SUs were randomly selected from each PU with a probability based on the size of their home. Following this enumeration, 10 households per SU were randomly selected as part of the third sampling stage. Once in the SU in question, the team leader updated the map of the SU and tallied all of the SU’s structures using the survey’s “computer-assisted personal interviewing” (CAPI) technology. The CAPI system then conducted systematic equal probability sampling to select 10 households in each cluster as a sample.

All women between the ages of 15 and 49 who regularly resided in the chosen houses or who had spent the night before the survey were eligible to participate in the study. All men between the ages of 15 and 59 who lived in five out of ten SU households were eligible to participate in the poll.

### Study population and selection criteria

All living children constituted the population for sampling. As part of the inclusion criteria, we focused on children below 6 months of age and living with their mothers. Additionally, only the last child in the past 2 years was considered. We realized a weighted sample size of 1,090.

### Study variables

#### Outcome variable

The MDHS collected data on the prevalence of breastfeeding from mothers using a 24-hour dietary recall method [29]. The dependent variable for this study was EBF for infants under six months of age. This was an indicator based on questions concerning the feeding of the child. Some of these questions include: gave child plain water, currently breastfeeding, gave child juice, milk, and solids. This is in sync with Chabé-Ferret [30] where a similar approach was utilised. Accordingly, all mothers who reported not providing their child with food other than breast milk in the 24 hours preceding the survey were assigned a code of 1, whereas those whose child had foods other than breast milk were coded 0.

#### Independent variables

The children’s ages were not grouped. The mother’s education level was classified as no education, primary, secondary/tertiary. Marital status was also regrouped as married or not married. Women who were categorised as not married included those who were divorced, widowed, separated and never married. Parity was categorised as 1, 2, 3, 4, 5, 6+. Working status was either working or not working. Antenatal care visits were recoded into no ANC, 1–3, 4+, and don’t know. Places of delivery were home, public health centre, and private health centre/other. Access to mass media was a combination of three variables: frequency of reading newspapers/magazines, frequency of listening to radio, and frequency of watching television. If an individual was frequent in at least one of them, we classified them as having access to mass media and vice versa. Other variables included in the study were the child’s sex, the mother’s age (years), place of residence (urban, rural), region and wealth. The household wealth index is a composite measure of household living standards, using measures such as ownership of assets, water access, sanitation, and materials used to build the house. A Principal Component Analysis (PCA) was applied, and the resulting scales were separated into five groups (poorest, poorer, middle, richer and richest) to form the wealth variable [2]. These variables were selected and recoded based on previous associations and a priori [31–36].

### Data analysis

We performed a logistic regression and presented the frequencies and bivariate analysis to provide the distribution of the variables considered for the study. A chi-squared analysis was also done to estimate whether there were significant differences between the outcome and independent variables or whether the differences were due to chance. We considered variables that were significant at the bivariate stage for the regression. We employed the logistic regression technique to establish the relationship between the binary dependent variable (EBF) and multiple independent variables. To improve the model parsimony and because of the study’s exploratory nature, adjustments were not made to the models [37]. Each regression model was subjected to an analysis of model fit performed using a backward stepwise selection method. The process starts with a model that includes all potential predictors and systematically removes variables one at a time based on their statistical significance or contribution to the model’s fit [38]. This helps simplify the model by removing unnecessary variables that may not contribute significantly to the outcome variable and reduces multicollinearity. This can result in a more interpretable and concise model. All estimations were weighted to ensure an accurate representation of the population. All statistics were completed using Stata Statistical Software (version 16, Stata Corp, TX, USA (2019). Based on the nature of the dataset, we used the corrected weighted Pearson chi-square statistics, and all results were weighted (using the individual weighting variable) to correct for over or understatements. A *p* < 0.05 was considered significant.

### Ethical considerations

The Measure DHS International Program granted permission to use the data set. Data was not shared with a third party and was only used for this study. All the data used in this investigation were anonymous, secondary, aggregated, and devoid of any personal identifiers. The dataset is fully available on the DHS website upon request [25].

## Results

Of a sample of 1,090, the prevalence of EBF among Mauritanian children who were less than 6 months old was 43.3% (Table 1). The highest proportion of children (39.5%) were within the ages of 3–4 months, and more than half were not exclusively breastfed. Although the sex of the child showed no significant difference with EBF, the results indicate 41.9% for males and 44.9% for females. Close to half (46.4%) of the mothers had a primary level of education. 24% (24.4%) of mothers were aged 25–29 years and the majority (95.3%) of the mothers were married. Almost a quarter of the mothers (24.9%) were among the poorest wealth category while the richest comprised 15.2%. Most (82.1%) of the mothers were not working and had 2–3 (45.8%) antenatal visits during pregnancy. 70.7% of mothers delivered in public health centres compared to 1.8% who delivered in private health centres/others. Mothers who indicated access to mass media usage such as television and radio were 63.3%. The highest proportion (58.7%) of mothers lived in rural areas and 16.5% in the Hodh Echargui region (Table 1).

**Table 1:** Frequency of exclusive breastfeeding practice among women in Mauritania

Variable	Freq (%)	Breastfeeding status		*P*-value
		Not exclusive	Exclusive	
		%[95%CI]	%[95%CI]	
**Exclusive breastfeeding status**				
Not exclusive	617(56.6)			
Exclusive breastfeeding	473(43.4)			
**Age of child (months)**				< 0.000
0–1	90(8.3)	34.2 [24.0-46.1]	65.8 [53.9–76.0]	
1	190(17.4)	39.6 [31.0–49.0]	60.4 [51.0–69.0]	
2	220(20.2)	54.0 [46.2–61.6]	46.0 [38.4–53.8]	
3	211(19.3)	56.9 [48.7–64.8]	43.1 [35.2–51.3]	
4	176(16.1)	67.3 [58.7–74.9]	32.7 [25.1–41.3]	
5	204(18.7)	75.5 [67.4–82.1]	24.5 [17.9–32.6]	
*Pearson: uncorrected chi2(5) = 79.1242*				
*Design-based f (4.88*,*1611.84) = 11.7128*				
**Sex of child**				*p* = 0.390
Male	545(50)	58.1 [53.0–63.0]	41.9 [37.0–47.0]	
Female	545(50)	55.1 [49.4–60.7]	44.9 [39.3–50.6]	
*Pearson: uncorrected chi2(1) = 0.9811*				
*Design-based f(1.00*,* 330.00) = 0.7404*				
**Maternal age (years)**				*p* = 0.080
15–19	149(13.7)	52.9 [41.9–63.7]	47.1 [36.3–58.1]	
20–24	215(19.7)	55.7 [48.2–62.9]	44.3 [37.1–51.8]	
25–29	266(24.4)	55.2 [47.5–62.7]	44.8 [37.3–52.5]	
30–34	216(19.9)	62.8 [54.4–70.5]	37.2 [29.5–45.6]	
35–39	161(14.8)	62.7 [53.0-71.4]	37.3 [28.6–47.0]	
40+	82(7.5)	41.5 [30.2–53.9]	58.5 [46.1–69.8]	
*Pearson: uncorrected chi2(5) = 14.4972*				
*Design-based f(4.75*,* 1566.88) = 1.9948*				
**Maternal education**				*p* = 0.021
No education	388(35.6)	53.3 [46.4–60.0]	46.7 [40.0-53.6]	
Primary	506(46.4)	54.7 [48.8–60.4]	45.3 [39.6–51.2]	
Secondary/higher	196(17.9)	68.2 [59.2–76.0]	31.8 [24.0-40.8]	
*Pearson: uncorrected chi2(2) = 13.2758*				
*Design-based f(1.99*,* 656.23) = 3.9024*				
**Marital status**				*p* = 0.861
Not married	51(4.7)	58 [42.1–72.3]	42 [27.7–57.9]	
Married	1039(95.3)	56.5 [52.3–60.7]	43.5 [39.3–47.7]	
*Pearson: uncorrected chi2(1) = 0.0408*				
*Design-based f(1.00*,* 330.00) = 0.0305*				
**Parity**				*p* = 0.285
One birth	208(19)	58.3 [49.3–66.8]	41.7 [33.2–50.7]	
Two births	198(18.1)	61.6 [52.5–70.0]	38.4 [30.0-47.5]	
Three births	162(14.9)	51 [42.4–59.5]	49 [40.5–57.6]	
Four births	144(13.2)	62.9 [53.1–71.7]	37.1 [28.3–46.9]	
Five births	123(11.2)	52.6 [42.2–62.8]	47.4 [37.2–57.8]	
Six or more births	256(23.5)	53.2 [46.1–60.2]	46.8 [39.8–53.9]	
*Pearson: uncorrected chi2(5) = 8.6415*				
*Design-based f(4.88*,* 1611.88) = 1.2474*				
**Wealth index**				< 0.000
Poorest	271(24.9)	50.9 [43.3–58.5]	49.1 [41.5–56.7]	
Poorer	257(23.6)	40.4 [33.2–48.0]	59.6 [52.0-66.8]	
Middle	194(17.8)	52.2 [44.5–59.7]	47.8 [40.3–55.5]	
Richer	203(18.6)	71.1 [63.7–77.5]	28.9 [22.5–36.3]	
Richest	165(15.2)	78.5 [68.8–85.8]	21.5 [14.2–31.2]	
*Pearson: uncorrected chi2(4) = 82.3667*				
*Design-based f(3.84*,* 1265.93) = 14.5837*				
**Maternal occupation**				*p* = 0.014
Not working	895(82.1)	54 [49.4–58.5]	46 [41.5–50.6]	
Working	195(17.9)	68.6 [58.2–77.4]	31.4 [22.6–41.8]	
*Pearson: uncorrected chi2(1) = 13.8607*				
*Design-based f(1.00*,* 330.00) = 6.1528*				
**Antenatal visit**				*p* = 0.302
No ANC	111(10.2)	54.8 [43.1–66.0]	45.2 [34.0-56.9]	
3-Jan	499(45.8)	53.4 [47.3–59.3]	46.6 [40.7–52.7]	
4 or more	416(38.2)	59.9 [54.1–65.3]	40.1 [34.7–45.9]	
Don’t know	65(5.9)	63.7 [48.6–76.4]	36.3 [23.6–51.4]	
*Pearson: uncorrected chi2(3) = 5.3873*				
*Design-based f(2.99*,* 985.61) = 1.2180*				
**Place od delivery**				*p* = 0.004
Home	301(27.6)	47.4 [39.5–55.4]	52.6 [44.6–60.5]	
Public health center	770(70.7)	59.6 [55.0-63.9]	40.4 [36.1–45.0]	
Private health center/other	19(1.8)	81.5 [52.8–94.5]	18.5 [5.5–47.2]	
*Pearson: uncorrected chi2(2) = 17.9390*				
*Design-based f(1.98*,* 654.01) = 5.6887*				
**Access to media**				*p* = 0.054
No	400(36.7)	52 [45.9–58.2]	48 [41.8–54.1]	
Yes	690(63.3)	59.2 [54.3–64.0]	40.8 [36.0-45.7]	
*Pearson: uncorrected chi2(1) = 5.3090*				
*Design-based f(1.00*,* 330.00) = 3.7387*				
**Type of place of residence**				< 0.000
Urban	451(41.3)	72.4 [66.9–77.3]	27.6 [22.7–33.1]	
Rural	639(58.7)	45.5 [40.5–50.6]	54.5 [49.4–59.5]	
*Pearson: uncorrected chi2(1) = 77.7967*				
*Design-based f(1.00*,* 330.00) = 47.7171*				
**Region**				< 0.000
Hodh Echargui	180(16.5)	42.9 [31.8–54.7]	57.1 [45.3–68.2]	
Hodh Gharbi	127(11.6)	52 [38.5–65.1]	48 [34.9–61.5]	
Assaba	90(8.3)	42.7 [33.9–52.1]	57.3 [47.9–66.1]	
Gorgol	104(9.5)	49.7 [39.8–59.7]	50.3 [40.3–60.2]	
Brakna	103(9.5)	49.5 [37.4–61.7]	50.5 [38.3–62.6]	
Trarza	58(5.3)	81.4 [68.6–89.8]	18.6 [10.2–31.4]	
Adrar	17(1.6)	58.8 [45.1–71.2]	41.2 [28.8–54.9]	
Dakhlet Nouadhibou	25(2.3)	46.7 [28.8–65.6]	53.3 [34.4–71.2]	
Tagant	26(2.3)	54.8 [41.4–67.6]	45.2 [32.4–58.6]	
Guidimagha	91(8.3)	38 [29.5–47.2]	62 [52.8–70.5]	
Tiris Zemour Et Inchiri	15(1.4)	85.8 [69.0-94.3]	14.2 [5.7–31.0]	
Nouakchott Ouest	58(5.3)	72.3 [54.5–85.0]	27.7 [15.0-45.5]	
Nouakchott Nord	90(8.3)	84.5 [72.1–92.0]	15.5 [8.0-27.9]	
Nouakchott Sud	106(9.8)	78.8 [65.0-88.2]	21.2 [11.8–35.0]	
*Pearson: uncorrected chi2(13) = 115.4629*				
*Design-based f(10.14*,* 3347.77) = 6.8941*				

### Factors associated with exclusive breastfeeding in Mauritania

Children aged 2 months were less likely (OR = 0.41; CI [0.20, 0.82]) to be EBF than those of 0 months. Interestingly, although children aged 3, 4 and 5 months were also less likely to be exclusively breastfed, the findings indicate a decrease in odds (38%, 17% and 13%) with age. Poorer mothers had an increased chance (OR = 2.13; CI [1.37, 3.31]) of breastfeeding exclusively than those in the poorest category. Mothers who were working were less likely (OR = 0.63; CI [0.41, 0.97]) to exclusively breastfeed their children. Residing in a rural area increased the likelihood (OR = 2.21; CI [1.46, 3.34]) of exclusively breastfeeding children compared to living in an urban area. The findings show that living in some regions was significant to EBF. For example, those who lived in the Nouakchott Nord region were less likely (OR = 0.29; CI [0.10, 0.85]) to breastfeed exclusively (Table 2).

**Table 2:** Factors associated with exclusive breastfeeding among women in Mauritania

Variables	Model 1	Model 2	Model 3
	OR[CI]	OR[CI]	OR[CI]
**Age of child (months)**			
0	Ref	Ref	Ref
1	0.78 [0.38,1.58]	0.78 [0.39,1.57]	0.78 [0.39,1.57]
2	0.41* [0.20.0.82]	0.41* [0.20.0.82]	0.41* [0.20.0.82]
3	0.38** [0.19,0.76]	0.38** [0.19,0.76]	0.38** [0.19,0.76]
4	0.17***[0.08,0.34]	0.17***[0.08,0.35]	0.17***[0.08,0.35]
5	0.13*** [0.06,0.27]	0.13*** [0.06,0.28]	0.13*** [0.06,0.28]
**Maternal educational level**			
No education	Ref		
Primary	1 [0.69,1.45]		
Secondary/higher	0.84 [0.50,1.41]		
**Wealth Index**			
Poorest	Ref	Ref	Ref
Poorer	2.09** [1.34,3.27]	2.08** [1.33,3.24]	2.13*** [1.37,3.31]
Middle	1.71* [1.02,2.88]	1.68* [1.01,2.81]	1.75* [1.06,2.90]
Richer	1.18 [0.63,2.20]	1.13 [0.61,2.10]	1.18 [0.65,2.15]
Richest	0.69 [0.30,1.56]	0.64 [0.29,1.41]	0.66 [0.31,1.41]
**Maternal Occupation**			
Not working	Ref	Ref	Ref
Working	0.64* [0.42,0.98]	0.64* [0.41,0.98]	0.63* [0.41,0.97]
**Place of delivery**			
Home	Ref	Ref	
Public health center	1.13 [0.77,1.67]	1.12 [0.76,1.66]	
Private health center/Other	0.89 [0.17,4.67]	0.85 [0.16,4.44]	
**Type of place of residence**			
Urban	Ref	Ref	Ref
Rural	2.19*** [1.42,3.37]	2.25*** [1.47,3.44]	2.21*** [1.46,3.34]
**Region**			
Hodh Echargui	Ref	Ref	Ref
Hodh Gharbi	0.6 [0.33,1.11]	0.6 [0.33,1.10]	0.6 [0.33,1.10]
Assaba	1.17 [0.64,2.15]	1.18 [0.64,2.16]	1.17 [0.64,2.15]
Gorgol	0.64 [0.34,1.22]	0.63 [0.33,1.20]	0.64 [0.34,1.22]
Brakna	0.58 [0.30,1.13]	0.58 [0.30,1.13]	0.59 [0.30,1.14]
Trarza	0.14*** [0.06,0.35]	0.15*** [0.06,0.35]	0.15*** [0.06,0.36]
Adrar	0.67 [0.29,1.54]	0.67 [0.29,1.52]	0.66 [0.29,1.51]
Dakhlet Nouadhibou	1.83 [0.58,5.76]	1.88 [0.60,5.91]	1.91 [0.61,5.95]
Tagant	0.52 [0.26,1.04]	0.51 [0.25,1.02]	0.51 [0.25,1.03]
Guidimagha	1.25 [0.69,2.30]	1.26 [0.69,2.30]	1.25 [0.69,2.28]
Tiris Zemour Et Inchiri	0.15** [0.04,0.54]	0.15** [0.04,0.55]	0.16** [0.04,0.55]
Nouakchott Ouest	0.74 [0.28,1.95]	0.76 [0.29,1.98]	0.75 [0.29,1.97]
Nouakchott Nord	0.28* [0.10,0.82]	0.29* [0.10,0.84]	0.29* [0.10,0.85]
Nouakchott Sud	0.5 [0.19,1.30]	0.52 [0.21,1.33]	0.53 [0.21,1.33]
N	1,090	1,090	1,090
pseudo R-sq	0.175	0.174	0.174
AIC	1340.22	1336.97	1333.59
BIC	1470.06	1456.83	1443.46

Exponentiated coefficients; 95% confidence intervals in brackets

* *p* < 0.05, ** *p* < 0.01, *** *p* < 0.001

## Discussion

This study aimed to identify the factors associated with EBF in Mauritania. The results show that the age of the infant, maternal working status, wealth index and place of residence were associated with EBF. The prevalence of EBF among Mauritanian children less than 6 months was 43.3% [0.41, 0.47].

Our findings show that EBF is socially patterned [39], with working status, geographical location and wealth status significantly associated with EBF at 6 months. Mothers who were not working practised EBF at a higher rate when compared to working mothers. The finding aligns with other studies [40, 41] showing that mothers in paid work often find it difficult to continue EBF after returning to work [42–44]. This may be associated with a lack of infrastructure supporting breastfeeding among working mothers such as paid parental leave, creches for working parents, and family-friendly workplace policies [45]. Mothers combining paid work with breastfeeding may be compelled to supplement breastfeeding with other foods. Moreover, the need for mothers to return to paid work to earn income compels many mothers to spend less time with their babies, especially as they age, hence the decline in EBF during the 4th to 6th month [46].

Consistent with other studies [11, 47–49], our study findings demonstrate that the practice of EBF, as expected, declines as the babies age. Social and cultural influences such as family and infant food practices and beliefs and physical breastfeeding issues such as low milk supply and pain play a role in the decline [50]. However, others have shown that targeted community and health services including postpartum care and support during the first 1–2 months after birth can extend EBF. In the Mauritanian context, as infants approach 6 months, grandparents and peers often assume that infants require more milk feeding for healthy growth, hence the introduction of supplementary feeding [43, 51, 52].

Our analysis showed that residing in a rural area was significantly associated with a higher prevalence of EBF when compared to mothers in urban areas. Interestingly, our finding is contrary to the findings of^,^ Mubratu and colleagues [53] where mothers living in rural areas were reported to be less likely to practice EBF due to poor nutritional diets required to produce breastmilk, limited access to health information and services, and cultural and religious beliefs [54]. Mothers in urban areas were reported to adhere to the practice of EBF more than rural mothers because of easy access to health information and services and the likelihood of being educated with a good source of income [11]. Nonetheless, evidence from other studies [55, 56] has also reported findings consistent with our results. This may be attributed to rural mothers’ lack of access to baby formula due to financial restraints or socio-cultural beliefs about baby formula, which may help to sustain exclusively breastfeeding.

Finally, the practice of EBF in our study was associated with the mother’s wealth index. Mothers who fell within the poorer wealth index status were significantly more likely to EBF their babies as compared to those even in the rich and richest wealth status. Awoke et al. [46] similarly, found that mothers who earned less income were more likely to exclusively breastfeed than mothers with a high monthly income in Ethiopia. Conversely, in high-income settings, the opposite phenomenon exists with high income being a positive factor in initiation and for the duration of EBF [39]. In the Mauritian context where infant formula costs US$6 or more on the average [57], this finding may suggest that the cost of baby formula makes it expensive for mothers within the low-income bracket to purchase [58].

### Strengths and limitations

The study made use of nationally representative data which provides a strength in being able to generalize the results to the population. We also used an appropriate methodological technique to identify associations. However, the study had some limitations. First, the study did not adjust for possible confounding variables which could affect the accuracy of the effects of the independent variables on the outcome. Moreover, the cross-sectional nature of the data does not permit causality but only shows associations. Also, the data was collected based on self-reported information, hence the likelihood of recall and social desirability biases. Lastly, given the cross-sectional nature of the study design, our ability to identify reasons behind some of the observations was limited, and issues such as the duration of breastfeeding cannot be measured using 24-hour dietary recall.

## Conclusion

Despite the 2025 national target and less than 5 years before the end of the 2030 SDGs, the proportion of infants exclusively breastfed remains low in Mauritania. The child’s age, household income index, maternal working status, region, and place of residence were associated with EBF practices. To enhance EBF, we recommend creating breastfeeding-friendly workplaces for employed mothers, including establishing on-site daycare centres for infants to enable mothers to juggle work and feeding with less stress. Also, extending maternity leave to a minimum of six months could promote EBF for working mothers. Furthermore, regions such as Trarza, Tiris Zemor Et Inchiri and Nouakchott Nord should enhance the implementation of maternal health clinics and health extension programmes to ensure pregnant women and breastfeeding mothers receive adequate health services and information.

## Data Availability

www.dhsprogram.com.
